# Instability in End-of-Life Goals and Preferences of Patients Who Are Seriously Ill: A Systematic Review

**DOI:** 10.1001/jamanetworkopen.2025.41264

**Published:** 2025-11-14

**Authors:** Shravya Murali, Louisa Camille Poco, Chetna Malhotra

**Affiliations:** 1 Health Services and Systems Research, Duke-NUS Medical School, Singapore; 2 Lien Centre for Palliative Care, Duke-NUS Medical School, Singapore

## Abstract

**Importance:**

Advance care planning (ACP) aims to ensure goal-concordant end-of-life (EOL) care based on patients’ prerecorded goals and preferences, assuming these remain stable. However, this assumption has not been systematically examined.

**Objective:**

To assess evidence on stability of EOL care goals and preferences over time among patients who are seriously ill, and identify factors associated with changes over time.

**Evidence Review:**

PubMed, EMBASE, and Scopus were searched for English-language longitudinal studies published between 2014 and September 2024 assessing outcomes at least twice among adults with a serious illness were included. Studies lacking sufficient data to calculate proportions of patients with unstable goals and preferences during the study period were excluded. Studies were assessed using dual independent screening, single-author data extraction and quality assessment with verification by second author. Study-level regression analyses and narrative synthesis were used to identify factors associated with instability.

**Findings:**

In this systematic review of 25 studies examining data from 5711 patients across 7 countries, 10 studies found that at least 50% of patients who are seriously ill changed EOL care goals and preferences over time. Fifteen studies were rated good quality, and 10 as fair quality. Study-level regression of studies rated as good quality using the National Institute of Health Quality Assessment Tool for Observational Studies found a higher number of assessments was significantly associated with greater observed instability (ie, a higher proportion of patients changing their goals or preferences (β = 0.07; 95% CI, 0.02 to 0.12). ACP intervention between assessments, time between assessments, follow-up duration, and patient illness (cancer vs noncancer) were not significantly associated. Narrative synthesis identified that changes in health status, emotional states, and prognostic understanding were key influences on instability. Prior ACP did not stabilize preferences over time.

**Conclusions and Relevance:**

In the studies included in this systematic review, EOL care goals and preferences among patients who are seriously ill were unstable and influenced by transient clinical and emotional factors. These shifts often go undetected when preferences were assessed infrequently. These findings suggest that ACP should be reframed—not as a future-focused plan for goal-concordant EOL care, but as an ongoing approach that prepares patients who are seriously ill and their families for real-time decisions in the face of uncertainty.

## Key Points

**Question:**

Are end-of-life care goals and preferences unstable among patients who are seriously ill?

**Findings:**

This systematic review across 25 studies found 10 studies with at least 50% of patients changing preferences over time. This instability is associated with number of assessments, and influenced by transient factors, such as health status, emotional states and prognostic understanding.

**Meaning:**

These findings suggest that end-of-life care goals and preferences among patients who are seriously ill are unstable; therefore care planning should focus on an ongoing process of preparing patients and families for real-time decision-making rather than relying on predocumented goals and preferences for future medical care decisions.

## Teaser

This systematic review assesses evidence about the stability of end-of-life goals and preferences among patients who are seriously ill and identifies factors that are associated with changes over time.

## Introduction

Advance care planning (ACP) supports end-of-life (EOL) care decision-making by encouraging individuals to share their EOL care goals and preferences with health care clinicians and caregivers.^1,2^ Traditionally, ACP’s main objective has been to align EOL care with communicated wishes.^1^ However, recent literature has questioned its effectiveness in consistently achieving this.^2,3,4,5,6,7^

These concerns stem from assumptions that patients’ prerecorded goals and preferences remain stable and thus applicable at the time of EOL decision-making. Earlier reviews based on studies with only 2 short interval assessments, supported this assumption.^8,9^ More recent research using multiple and longer-term assessments indicate that EOL care goals and preferences change over time.^10,11^ Behavioral decision theories, such as projection bias—the tendency to assume current preferences will persist despite changes in health, emotions, and prognostic understanding—help explain this instability.^3,11,12,13,14,15,16^

Previous reviews on preference stability also overlooked changes in broader goals for care—the overarching objectives for medical care shaping preferences for specific interventions.^8,9,17,18,19^ Since goals-of-care discussions are central to ACP and serious illness conversations, understanding how these goals evolve over time is critical for informing ACP practice globally.

Beyond changing health states and prognostic understanding, several factors may influence instability. While some studies link prior ACP to greater stability,^9^ others indicate that ACP may prompt reflection and change in goals or preferences.^20,21^ In particular, cancer patients may exhibit greater instability due to their unique emotional and treatment trajectories.^22,23^ Understanding these influences is key to optimizing ACP implementation.

This systematic review examined whether the EOL care goals and preferences change over time among patients who are seriously ill and hypothesized that they were unstable. We also assessed study-level characteristics, hypothesized that studies involving predominantly cancer patients, more frequent assessments, longer assessment periods or intervals, and ACP interventions between assessments, will report greater instability. Lastly, we synthesized within-study (patient-level) factors influencing preference instability, such as patients’ health and emotional states, prognostic understanding, initial preferences, prior ACP, ACP intervention between assessments, and changes in broader goals of care.

## Methods

This systematic review used only previously published data and was exempt from institutional review board approval per the Common Rule. It followed the relevant recommendations of the Preferred Reporting Items for Systematic Reviews and Meta-Analyses (PRISMA) reporting guideline^24^ and is registered in PROSPERO (CRD42024628488).

### Outcome

The outcome assessed was instability in patients’ EOL care goals and preferences, defined as any change occurring at least once during the study period. EOL care refers to care provided in the final months or years of life.^25,26^ Preferences included patients’ stated choices regarding life-sustaining treatments (eg, intensive care, cardiopulmonary resuscitation, mechanical ventilation), and place of care or death^27^; nonmedical preferences (e,g decision-making role) were excluded. Goals of care referred to overarching medical objectives (eg, extending life, reducing pain) reflecting core beliefs about living well.

### Eligibility Criteria

We included longitudinal studies (with or without interventions) assessing outcomes at least twice, reported by adults (aged 18 years or older) with serious illnesses—conditions with high mortality risk and substantial symptom burden, impaired quality of life or functional decline.^28^ To avoid overlap with previous systematic reviews, we included only English-language studies that were published after 2014.^8,9^ We excluded cross-sectional studies, systematic reviews, meta-analyses, case reports, conference abstracts, narrative reviews, editorials, studies focused on children, acute illnesses, general populations, clinicians or caregivers. We also excluded studies that did not report the proportion of patients whose goals or preferences changed over time. We did not limit studies by duration between outcome assessments, acknowledging that patients’ goals and preferences may change over shorter periods.^29^

### Search Strategy

We searched PubMed, Embase, and Scopus with a combination of Medical Subject Headings and keywords, such as *end-stage*, *change*, *patient preference*, and *goals*, following the recommendations of Peer Review of Electronic Search Strategies.^30^ The search covered publications from January 1, 2014, to September 13, 2024 (eTable 3 in Supplement 1).

### Study Selection, Data Extraction, and Quality Assessment

References were imported to EndNote (Microsoft) for duplicate removal. Rayyan was used for further deduplication, followed by primary and secondary screening of the remaining articles. Two reviewers (S.M. and L.R.P.) independently screened titles, abstracts, and full-texts, and resolved disagreements through discussions and senior author adjudication. Data were extracted into an Excel (Microsoft) template, including study details (authors, publication year, country of origin, study aim, setting, sample size), patient characteristics (age, gender, ethnicity, type of illness, prior ACP exposure), mode, number and timing of data collection, goals or preferences elicited, ACP between assessments, and study conclusions. The numbers and proportions of patients whose goals or preferences changed were recorded, with the highest reported proportion used when multiple estimates were given.

ACP was defined as any intervention prompting patients to consider, reflect on, and communicate their EOL care goals or preferences for current and future medical care. We assessed study quality using the 14-item National Health Institute’s (NIH) quality assessment tool for observational cohort studies.^31^ Items were scored as yes (ie, 1), no or not reported (ie, 0), or not applicable (ie, excluded), with partial satisfaction scored 0.5. Overall ratings were good (75% or more), fair (50% to 75%), or poor (less than 50%)^32^ (eAppendix in Supplement 1). Data extraction and quality ratings by the primary reviewer (S.M.) were cross-checked by the secondary reviewer (L.R.P.), with disagreements resolved through discussions and senior author review.

### Statistical Analyses

We summarized study characteristics using bar charts. Where possible, we examined the natural trajectory of changes in goals and preferences. For intervention studies reporting outcomes separately for ACP intervention and control groups, only control group data were analyzed. In pre-post designs, we included all participants.

We constructed bubble charts depicting the proportion of patients with 1 or more change in goals or preferences against number of assessments, labeled by disease-type (cancer vs noncancer), for all studies and for good-quality studies only.

Among good-quality studies, we conducted a simple ordinary least-squares linear regression to examine associations between study-level characteristics (number of assessments, time between assessments, follow-up duration, presence of predominantly cancer populations [ie, populations that included 50% or more patients with cancer], and ACP intervention between assessments) and the proportion of patients with unstable goals or preferences.^33^ For control group–only studies, we assumed no ACP intervention.

Lastly, we performed a narrative synthesis exploring influence of patient-level (within-study) factors including prior ACP, health and emotional states, and prognostic understanding. Factors were considered significant if *P* < .05. All analyses were performed using Excel version 16.1 (Microsoft) and Stata version 14 (StataCorp).^34^ Data were analyzed from January to March 2025. 

## Results

**Figure 1.**
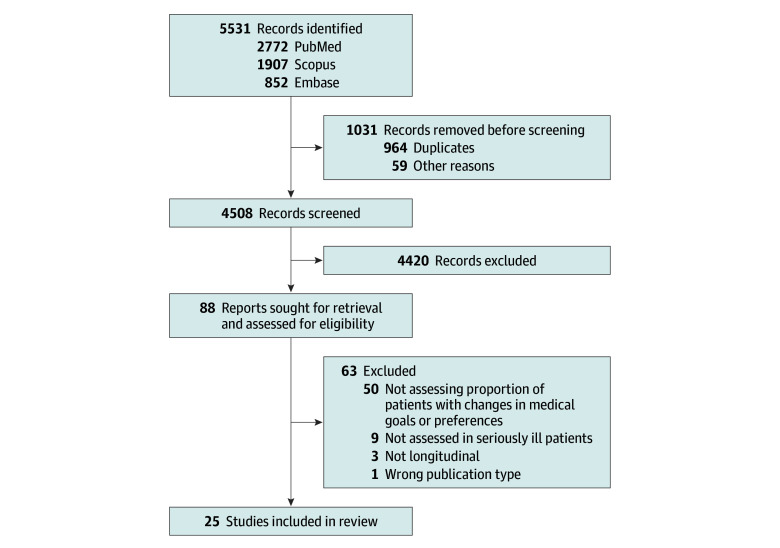
PRISMA Flow Diagram

We screened 4508 titles and abstracts, including 25 studies,^10,11,16,22,23,35,36,37,38,39,40,41,42,43,44,45,46,47,48,49,50,51,52,53,54^ representing 5711 patients (Figure 1). The mean (SD) age of participants across studies ranged from 32.6 (6.0) years to 88.7 (7.9) years, and 2993 of 5711 (52.4%) were males. One study was excluded due to unavailable information on proportions of patients changing their goals.^55^ Some studies represented multiple publications from the same parent trials: (1) 2 were from a trial of advanced heart failure patients in Singapore, examining different outcomes—preferences for life-sustaining treatments and place of death^10,46^; (2) 2 studies from a US trial examining overall goals of care with different time points and sample sizes^37,53^; and (3) 2 studies using data from the same pilot trial of young adults with advanced cancer, with different patient samples.^41,48^

### Study Characteristics

Among the 25 studies, 13 (52%) were conducted in the US,^22,23,35,37,41,43,44,47,48,49,52,53,54^ 4 (16%) in Netherlands,^11,38,42,50^ 3 (12%) in Singapore,^10,16,46^ 2 (8%) in Canada,^36,45^ 1 (4%) in Germany,^40^ 1 (4%) in Brazil,^39^ and 1 (4%) in South Korea.^51^

Seventeen studies (68%) studies focused on specific serious illnesses—11 (44%) on advanced cancer,^11,16,39,40,41,43,48,50,51,52,54^ 4 (16%) on heart failure,^10,36,44,46^ and 2 (8%) on kidney failure^47,49^—while 8 (32%) included mixed illnesses.^22,23,35,37,38,42,45,53^ Overall, 13 studies (52%) focused predominantly on cancer patients.^11,16,22,35,39,40,41,43,48,50,51,52,54^ Ten studies (40%) administered ACP interventions between assessments.^10,35,36,44,46,48,49,52,53,54^ For 2 studies (8%), only data from the control arms—without ACP intervention—were included in our review.^47,51^

Most studies (19 [76%]) used questionnaires^10,16,37,38,39,40,41,42,43,44,46,47,48,49,50,51,52,53,54^; remainder used medical records (4 [16%])^22,23,35,36^ or both (2 [8%]).^11,45^ Outcomes included goals of care (10 [40%]),^11,22,36,37,41,43,48,50,53,54^ life sustaining treatment preferences (10 [40%]),^10,23,35,40,42,45,47,49,51,52^ place of death (4 [16%])^16,38,39,46^ and both goals of care and life sustaining treatment preferences (1 [4%])^44^ (eTable 4 in Supplement 1).

### Number, Time Between Assessments, and Duration of Follow-Up 

**Figure 2.**
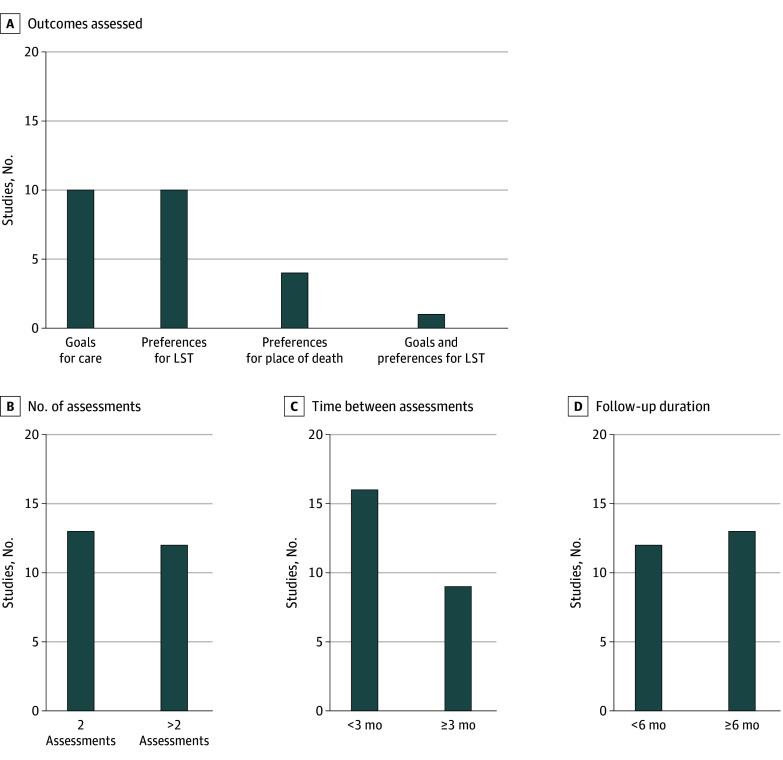
Outcomes, Number, Time Between Assessments, and Duration of Follow-Up LST indicates life-saving techniques.

The number of outcome assessments ranged from 2 to 7; 12 studies (48%) had more than 2 assessments.^10,11,16,38,39,42,43,44,46,50,51,54^ Time between the first 2 assessments ranged from immediate to 6 months; 9 studies (36%) conducted assessments at least 3 months apart (Figure 2).^10,16,22,37,39,40,41,42,46^ Follow-up duration ranged from immediate to 6 years, with 13 studies (52%) conducted over at least 6 months.^10,11,16,22,36,38,39,40,42,44,46,50,54^

### Methodological Quality

Fifteen studies (60%) were rated good,^10,11,16,23,35,36,37,44,45,46,47,48,49,51,53^ and 10 (40%) fair (eTable 5 in Supplement 1).^22,38,39,40,41,42,43,50,52,54^ Common weaknesses included loss to follow-up, lack of sample size justification, inadequate adjustment for confounders, and incomplete exposure assessment across time points among studies.

### Changes in End-of-Life Care Goals or Preferences and Study-Level Regression

**Figure 3.**
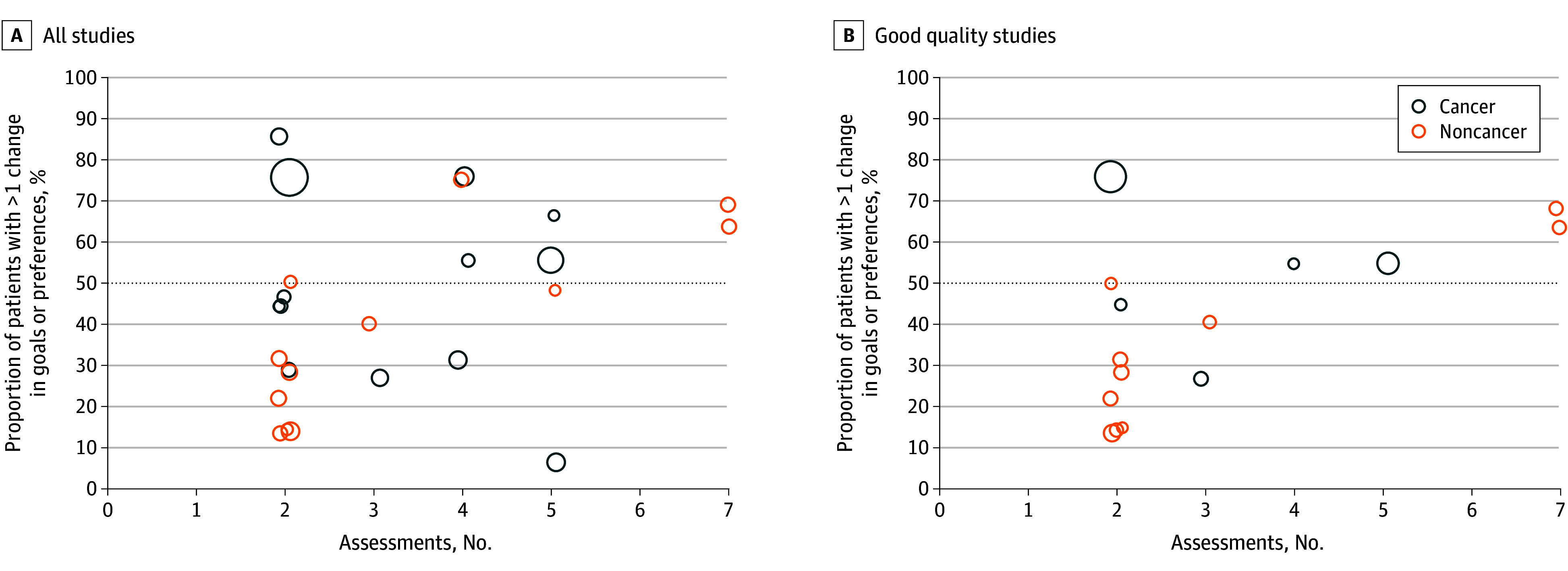
Changes in End-of-Life Care Goals or Preferences by Number of Assessments, and Disease Type

Across all studies, 10 (40%) reported more than half of patients had unstable goals or preferences^10,11,16,22,35,42,46,49,50,54^; among the 15 good quality studies, 6 (40%) met this threshold.^10,11,16,35,46,49^ Studies with only 2 assessments showed lower proportions of patients with unstable goals or preferences (Figure 3).

Study-level regression among good quality studies further showed that a higher number of assessments (β = 0.07; 95% CI, 0.02 to 0.12) was associated with a higher proportion of patients changing their goals or preferences (Table 1). ACP intervention between assessments, time between assessments, follow-up duration, and patient illness (cancer vs noncancer) were not significantly associated.

**Table 1.** Univariable Study-Level Regression Among Good-Quality Studies on the Proportions of Patients With Unstable Goals or Preferences (N = 15)

Study-level factors	Coefficient (95% CI)	*P* value
Variables		
Time between assessments, mo	0.05 (−0.01 to 0.11)	.12
Number of assessments	0.07 (0.02 to 0.12)^a^	.02
Follow-up duration, mo	0.00 (−0.006 to 0.007)	.85
ACP intervention between assessments		
No ACP intervention between assessments	1 [Reference]	NA
ACP intervention between assessments	0.20 (−0.008 to 0.41)	.06
Disease type		
Non-cancer	1 [Reference]	NA
Cancer	0.17 (−0.06 to 0.40)	.14

Abbreviations: ACP, advance care planning; NA. not applicable.

^a^ *P* < .05.

### Narrative Synthesis of Patient-Level Factors

#### Health Status

Six studies (24%) (2 randomized controlled trials [RCTs],^11,38,46^ 1 prospective,^16^ and 2 retrospective cohort studies^22,23^) found health status—including well-being, recent hospitalization, and cancer stage—associated with changes in goals or preferences (Table 2). One study indicated that patients with poor well-being were less likely to change their preferences,^38^ while another reported they were more likely to change preferences to an institutional death,^46^ Recently hospitalized patients were likely to change preferences to a home death.^16^ Two studies found cancer (vs noncancer) patients were more likely to change their goals^22^ or preferences,^23^ although the direction of change was unspecified. Another study suggested that a late-phase cancer patients changed their goals more frequently than early-phase patients although statistical significance was not tested.^11^

**Table 2.** Patient-Level Factors Examined in Narrative Synthesis

Domain	Factors	Total No. of studies	No. of studies in which factors were significantly associated	Direction of associations
Health status	Poorer well-being	2^38,46^	2^38,46^	One indicated more likely to change preferred place of death to an institution^46^, direction unspecified in the other study^38^
	Recent hospitalization	1^16^	1^16^	More likely to change preference to home death
	Cancer diagnosis	2^22,23^	2^22,23^	Direction unspecified
	Cancer stage	1^11^	NA (not tested)	Direction unspecified
Emotional states	Hopelessness	1^50^	1^50^	More likely to change goals toward improving quality of life
	Depression	1^44^	1^44^	Direction unspecified
	Psychological distress	1^16^	1^16^	More likely to change toward preferring either home or institutional death
Prognostic understanding	Accurate prognostic understanding	4^10,16,43,46^	3^10,43,46^	Inaccurate prognostic understanding more likely to change toward preferences for aggressive care in 1 study^10^
	Higher illness acceptance	1^44^	1^44^	Direction unspecified
ACP	Preexisting ACP at baseline	2^16,40^	1^16^	Direction unspecified
	ACP between assessments	4^10,44,46,49^	0	NA
Initial EOL preferences	Preferences at baseline	2^16,38,40,54^	2^38,54^	Initial unclear place of death preference associated with changes, while initial home death preferences less likely to change in 1 study^54^
Goals for EOL care	Instability in EOL care goals	1^44^	1^44^	Direction unspecified
Demographics	Age	9^10,16,22,23,42,43,44,46,50^	0	NA
	Gender	8^10,16,22,23,42,43,44,46,50^	0	NA
	Ethnicity	4^10,16,43,46^	2^10,16^	Malay patients less likely that Chinese patients to change toward preferring institutional death in one study^16^; non-Chinese and non-Malay patients more likely to change preferences to aggressive care in another study^10^
	Education level	5^10,16,43,46,50^	1^50^	Lower education associated with changing goals toward life-prolongation

Abbreviations: ACP, advance care planning; EOL, end-of-life; NA, not applicable.

#### Emotional States

Three studies (12%) (1 observational study^16^ 2 RCTs^44,50^) found a significant association between emotional states and change in goals and preferences for EOL care.^16,44,50^ One reported helplessness or hopelessness being associated with changes in goals toward improving quality of life over life extension.^50^ Another reported depression as being associated with changes in goals of care (extending life vs living comfortably), though the direction of change was not specified.^44^ Furthermore, a study found that patients with psychological distress were more likely to change preferred place of death, either to home or to an institution.^16^

#### Prognostic Understanding

Five studies (20%) (2 observational studies^16,43^ and 3 RCTs^10,44,46^) assessed prognostic understanding (patients’ awareness of their curability or disease stage)^10,16,44,46^ or illness acceptance.^43^ Four studies found significant associations.^10,43,44,46^ Three studies found that patients with inaccurate prognostic understanding were more likely to shift preferences toward aggressive care,^10^ change their preferred place of death,^46^ or change preferences for life-prolonging or comfort care.^43^ Another study reported patients with lower illness acceptance being more likely to change their goals.^44^

### ACP

#### Prior or Preexisting ACP at Baseline

Two prospective cohort studies (8%)^16,40^ examined associations between having ACP or AD documented before study enrolment and stability or instability of goals and preferences. One study reported that having a preexisting ACP before study enrolment increased the likelihood of patients changing their preferences for place of death during the study period,^16^ whereas the other study reported no association between having an ACP and changes in patient goals or preferences.^7^

#### ACP Between Assessments

Three RCTs (12%)^10,44,46^ found no significant association between ACP intervention^10,44,46^ and change in preferences. A fourth RCT^49^ found no difference in preference instability across ACP types (ACP vs AD).

### Initial EOL Preferences

Two studies (8%) (1 prospective cohort^54^ and 1 RCT^38^) showed initial preferences influenced instability; patients without clear baseline preferences were most likely to change, while those preferring home death were least likely.^38^ Patients with lower-intensity baseline treatment preferences were also more likely to change their treatment goals.^54^

### Association Between Goals and Preferences

One RCT (4%)^44^ reported changes in goals of care (extending life vs living comfortably) were associated with changes in specific treatment preferences (for left ventricular assist devices), although direction was unspecified.

### Demographic Factors

Nine studies (36%) (4 RCTs,^10,44,46,50^ 3 prospective,^16,42,43^ and 2 retrospective cohort studies^22,23^) examined demographics. None found age and gender to be associated with changes in EOL care goals or preferences.

Evidence for ethnicity and education was mixed. Of 4 studies (2 RCTs^10,46^ and 2 prospective cohort studies^16,43^), 1 prospective cohort study found Malay patients with cancer were less likely than Chinese patients to change preferences to an institutional death,^16^ and one RCT found that non-Chinese/non-Malay patients with heart failure were more likely to change their preference to aggressive EOL care.^10^ Among 5 studies examining education (2 prospective cohort studies^16,43^ and 3 RCTs^10,46,50^), only 1 RCT reported lower education patients were more likely to change their goals toward life prolongation.^50^

## Discussion

Our systematic review demonstrates that EOL care goals and preferences among patients who are seriously ill are dynamic, challenging previous conclusions of preference stability.^8,9^ We found no difference in rates of change between goals and preferences, indicating neither is inherently more stable, and that shifts in one coincide with shifts in the other. These findings support that providing goal-concordant care solely through ACP documentation may be elusive.

We found that studies with a higher number of assessments were more likely to capture changes in EOL care goals and preferences. This underscores the highly dynamic nature of EOL care goals and preferences—not easily captured through infrequent assessments. For example, a study (not included in our review) assessing terminally ill patients’ will-to-live every 12 hours reported that their preferences fluctuated with changes in health and emotional states.^29^ Conversely, another study with only 2 assessments over 6 years found that less than one-fourth of patients changed their EOL care goals.^36^

Interestingly, while our study-level regression found no significant differences in instability between cancer and noncancer studies—likely due to between-study heterogeneity—2 studies in our narrative review reported higher instability among cancer patients.^22,23^ This may reflect the treatment-intensive and emotionally turbulent trajectory of cancer care, as patients alternate between hope when receiving treatment and a lack of purpose after treatment. This contrasts with the more gradual course and acceptance-based orientation common in other serious illnesses,^56,57^ contributing to more stability in their goals and preferences. Our narrative synthesis also supports that the timing, such as a later cancer stage, and significant events, such as hospitalisation, along the disease trajectory, may influence instability in goals and preferences.^11,16^

Our narrative synthesis identified that changes in health status, emotional states, and prognostic understanding were key drivers of changes in patients’ goals and preferences, aligning with prior reviews.^8,9^ However, contrary to our hypothesis and findings from prior reviews, there was unclear evidence that prior or preexisting ACP or ACP intervention between assessments influenced the stability or instability of goals and preferences. This likely reflects differences in study populations: patients who are seriously ill face imminent, complex, and emotionally challenging decisions, with health, emotions, and prognostic understanding changing rapidly. As a result, preexisting ACP or AD documentation may not prevent shifts in care preferences, and preferences can change regardless of ACP interventions.

### Strengths and Limitations

The main strength of our review is that we examined both goals and preferences —offering a more comprehensive perspective than prior reviews—and restricted study-level regression to high-quality studies, enhancing the robustness of our conclusions. However, our review also has some limitations. We excluded studies lacking sufficient data to calculate the prevalence of instability, limiting the comprehensiveness of our review. Moreover, a few included studies classified patients lost to follow-up as having stable preferences, potentially underestimating the prevalence of instability. Another limitation was that we were unable to account for ACP interventions that may have occurred as part of routine care but were not reported in the included studies. Furthermore, the majority of studies included in our review are from North America. However, while goals and preferences for care may be shaped by culture, we found no clear evidence in the literature or in our narrative synthesis that cultural factors affect the likelihood of patients changing their goals and preferences.^58^ Finally, considering the small number of studies included in our regression analyses, our results should be treated as exploratory.^59^

## Conclusions

Overall, in this systematic review of 25 studies, EOL care goals and preferences among patients who are seriously ill changed frequently. Instability was driven by changes in health, emotional states, and prognostic understanding—and became more evident with frequent assessments. Crucially, ACP does not necessarily reduce this instability. Even repeated ACP discussions may not be able to capture patients’ evolving goals and preferences. These findings underscore the need to reframe ACP—not as a means to achieve goal-concordant care, but as a dynamic, ongoing process of preparing patients and families for real-time, in-the-moment decision-making.

## Notes

Supplement 1. **eTable 1. **Complete Online Strategies **eAppendix. **Items in Quality Assessment Tool **eTable 2. **Study Characteristics **eTable 3. **Rating of Study Quality
eReferences

Supplement 2.
Data Sharing Statement
