# Supplementary material for: Instability in End-of-Life Goals and Preferences of Patients Who Are Seriously Ill: A Systematic Review
Source: JAMA Netw Open. 2025 Nov 14;8(11):e2541264. doi: 10.1001/jamanetworkopen.2025.41264 (PMC12619100; doi:10.1001/jamanetworkopen.2025.41264)
Supplement: Supplement 1. — eTable 1. Complete Online Strategies eAppendix. Items in Quality Assessment Tool eTable 2. Study Characteristics eTable 3. Rating of Study Quality eReferences [file jamanetwopen-e2541264-s001.pdf]

Supplemental Online Content

Murali S, Poco LCR, Malhotra C. Instability End-of-Life goals and preferences of seriously ill patients. *JAMA Netw Open*. 2025;8(11):e2541264. doi:10.1001/jamanetworkopen.2025.41264

eTable 1. Complete Online Strategies

eAppendix. Items in Quality Assessment Tool

eTable 2. Study Characteristics

eTable 3. Rating of Study Quality

eReferences

This supplemental material has been provided by the authors to give readers additional information about their work.

© 2025 Murali S et al. *JAMA Network Open*.

© 2025 Murali S et al. *JAMA Network Open*.

eTable 1. Complete online strategies

Database	Search strategy
**PubMed (2772)**	((((((((((((((((change) OR (stability)) OR (stable)) OR (consistent)) OR (consistency)) OR (inconsistent)) OR (inconsistency)) AND ((((((((longitudinal) OR (observational)) OR (prospective cohort)) OR (cohort)) OR (case control)) OR (case-control)) OR (clinical trial)) OR (controlled trial)) OR (follow-up)) AND ((((((((((((((((((((((((((("place of death") OR ("place of care")) OR (home)) OR (hospital)) OR (hospice)) OR (symptom relief)) OR (life-sustaining)) OR (life extension)) OR (extending life)) OR (reduce pain)) OR (maintaining independence)) OR ("code status")) OR (patient comfort[MeSH Terms])) OR ("intensive care")) OR (hemodialysis)) OR ("blood transfusion")) OR (tube feeding)) OR (intubation)) OR ("mechanical ventilation")) OR (resuscitation)) OR (do-not-resuscitate)) OR (palliative chemotherapy)) OR (palliative surgery)) OR ("Implantable cardioverter defibrillator")) OR ("Ventricular Assist Devices")) OR ("Cardiac resynchroni*ation therapy")) OR (transplant)) OR ("oxygen therapy")) AND ((((((((((((((((((((((((((((advanced) OR (incurable)) OR (metastatic)) OR (terminally ill[MeSH Terms])) OR (end stage)) OR (end-stage)) OR (stage IV cancer)) OR (stage 4 cancer)) OR (heart failure)) OR (severe dementia)) OR (advanced dementia)) OR (end-stage dementia)) OR ("Chronic obstructive pulmonary disease")) OR (kidney failure)) OR (renal failure)) OR (organ failure)) OR (end-of-life)) OR (end of life)) OR (terminal care[MeSH Terms])) OR (palliative care[MeSH Terms])) OR (palliative)) OR (non-curable)))) OR (life-sustaining)) OR ("serious illness")) OR ("seriously ill")) OR ("life threatening")) OR (advanced)) OR (life-limiting)) AND (((patient preference[MeSH Terms]) OR (goals[MeSH Terms])) OR (Preference) OR wishes)<br>Additional filters: Articles published from 2014 to 2024
**Embase (852)**	(advanced OR incurable OR 'non curable' OR metastatic OR 'terminally ill patient'/exp OR ‘terminal disease’ OR ‘life limiting condition’ OR ‘life limiting illness’ OR ‘serious illness’ OR ‘seriously ill’ OR ‘life sustaining treatment’ OR ‘life threatening’ OR ‘life threatening illness’ OR 'end stage' OR 'stage iv cancer' OR 'stage 4 cancer' OR 'heart failure'/exp OR 'severe dementia' OR 'advanced dementia' OR 'end-stage dementia' OR 'chronic obstructive lung disease'/exp OR 'kidney failure'/exp OR 'organ failure' OR 'palliative care' OR 'end of life' OR 'terminal care'/exp OR palliative) AND (change OR stability OR stable OR consistent OR consistency OR consistencies OR inconsistent OR inconsistency OR inconsistencies) AND ('patient preference'/exp OR preference OR goal OR wish) AND ('place of death'/exp OR 'place of care' OR home OR hospital OR 'symptom relief' OR 'life sustaining' OR 'life extension' OR 'extending life' OR 'reduce pain' OR 'maintaining independence' OR 'code status' OR 'patient comfort'/exp OR 'intensive care'/exp OR 'hemodialysis'/exp OR 'blood transfusion'/exp OR 'feeding tube'/exp OR 'artificial ventilation'/exp OR 'resuscitation'/exp OR 'do not resuscitate order'/exp OR 'palliative chemotherapy'/exp OR 'palliative surgery' OR 'implantable cardioverter defibrillator'/exp OR 'ventricular assist device'/exp OR 'cardiac resynchronization therapy'/exp OR 'transplantation'/exp OR 'oxygen therapy'/exp) AND ('longitudinal study'/exp OR observational OR cohort OR 'case control' OR 'case control study'/exp OR 'clinical trial'/exp OR 'controlled trial' OR 'follow up'/exp) AND (2014:py OR 2015:py OR 2016:py OR 2017:py OR 2018:py OR 2019:py OR 2020:py OR 2021:py OR 2022:py OR 2023:py OR 2024:py) AND 'Article'/it
**Scopus (1907)**	(TITLE-ABS-KEY (advanced OR incurable OR non-curable OR metastatic OR (terminal* AND ill) OR (terminal AND illness) OR (serious* AND ill) OR (serious AND illness) OR end-stage OR life-sustaining OR life-threatening OR life-limiting OR "stage IV cancer" OR "stage 4 cancer" OR (heart AND failure) OR (severe AND dementia) OR (advanced AND dementia) OR (end-stage AND dementia) OR "Chronic obstructive pulmonary disease" OR (kidney AND failure) OR (renal AND failure) OR (organ AND failure) OR end-of-life OR (terminal AND care) OR (palliative AND care) OR palliative) AND TITLE-ABS-KEY (change OR stability OR stable OR consistent OR consistency OR consistencies OR inconsistent OR inconsistency OR inconsistencies) AND TITLE-ABS-KEY (patient AND preference OR preference OR goals OR wishes) AND ALL ("place of death" OR "place of care" OR

© 2025 Murali S et al. *JAMA Network Open*.

	home OR hospital OR hospice OR (symptom AND relief) OR life-sustaining OR (life AND extension) OR (extending AND life) OR (reduce AND pain) OR (maintaining AND independence) OR (code AND status) OR (patient AND comfort) OR "intensive care" OR hemodialysis OR (blood AND transfusion) OR (tube AND feeding) OR intubation OR "mechanical ventilation" OR resuscitation OR do-not-resuscitate OR (palliative AND chemotherapy) OR (palliative AND surgery) OR "implantable cardioverter defibrillator" OR (ventricular AND assist AND device) OR "cardiac resynchroni?ation therapy" OR transplant OR "oxygen therapy") AND TITLE-ABS-KEY (longitudinal OR observational OR cohort OR case-control OR (clinical AND trial) OR (controlled AND trial) OR follow-up)) AND PUBYEAR > 2013 AND PUBYEAR < 2025 AND PUBYEAR > 2013 AND PUBYEAR < 2025 AND (LIMIT-TO (DOCTYPE , "ar")) AND (LIMIT-TO (LANGUAGE , "English"))

© 2025 Murali S et al. *JAMA Network Open*.

eAppendix. Items in quality assessment tool

Quality of included studies were assessed based on the following criteria in the NIH quality assessment tool:

1. Was the research question or objective in this paper clearly stated?

2. Was the study population clearly specified and defined?

3. Was the participation rate of eligible persons at least 50%?

4. Were all the subjects selected or recruited from the same or similar populations (including the same time period)? Were inclusion and exclusion criteria for being in the study prespecified and applied uniformly to all participants?

5. Was a sample size justification, power description, or variance and effect estimates provided?

6. For the analyses in this paper, were the exposure(s) of interest measured prior to the outcome(s) being measured?

7. Was the timeframe sufficient so that one could reasonably expect to see an association between exposure and outcome if it existed?

8. For exposures that can vary in amount or level, did the study examine different levels of the exposure as related to the outcome (e.g., categories of exposure, or exposure measured as continuous variable)?

9. Were the exposure measures (independent variables) clearly defined, valid, reliable, and implemented consistently across all study participants?

10. Was the exposure(s) assessed more than once over time?

11. Were the outcome measures (dependent variables) clearly defined, valid, reliable, and implemented consistently across all study participants?

12. Were the outcome assessors blinded to the exposure status of participants?

© 2025 Murali S et al. *JAMA Network Open*.

13. Was loss to follow-up after baseline 20% or less?

14. Were key potential confounding variables measured and adjusted statistically for their impact on the relationship between exposure(s) and outcome(s)?

© 2025 Murali S et al. *JAMA Network Open*.

eTable 2. Study characteristics

Source, year	Country	Study design	Age[a]	Gender[a]	Ethnicity[a]	Type of illness	Sample size[b]	Mode of data collection	Prior exposure to ACP/AD/other forms of EOL care planning or discussions	ACP intervention between assessments (if any)
Auriemma et al., 2024[1]	USA	Retrospective cohort study (chart review)	Median: 70; IQR: 63-79	Not reported	Not reported	Conditions with >=50% risk of death within 6 months from admission	77	Electronic medical records	Not reported	Not applicable
Benton et al., 2015[2]	USA	Retrospective cohort study (chart review)	18-65 – 32.9%; >65 – 62.8%; Unknown – 4.3%	Male - 47.9%; Female - 51.8%; Unknown - 0.3%	White - 50.4%; Black - 35.0%; Unknown - 14.6%	Life-limiting illness (cancer and non-cancer)	2071	Medical records	100.0% (code status documentation)	Standardized counselor-based palliative care consultation
Campos et al., 2022[3]	Canada	Retrospective cohort study	Mean: 88.7; SD: 7.9	Male - 46.4%; Female - 53.6%	Not reported	Referred to palliative care for advanced heart failure defined according to 2021 ESC guidelines<br><br>(69.0% of patients had New York Heart Association class III or IV advanced HF symptoms)	250	Electronic medical records	Not reported	Collaborative home-based palliative care

© 2025 Murali S et al. *JAMA Network Open*.

Source, year	Country	Study design	Age[a]	Gender[a]	Ethnicity[a]	Type of illness	Sample size[b]	Mode of data collection	Prior exposure to ACP/AD/other forms of EOL care planning or discussions	ACP intervention between assessments (if any)
Curtis et al., 2018[4]	USA	Randomised controlled trial	Mean: 73.5; SD: 12.6	Male - 52.4%; Female - 47.6%	White non-hispanic - 79.1%; Ethnic minority - 20.9%	Metastatic cancer or inoperable lung cancer; or COPD with forced expiratory volume in 1 second (FEV1) values below 35% of that predicted or oxygen dependence, restrictive lung disease with a total lung capacity below 50% of that predicted, or cystic fibrosis with FEV 1 below 30% of that predicted; or New York Heart Association class III or IV heart failure, pulmonary arterial hypertension with a 6-	277	Telephone or mailed questionnaire	Not reported	Not applicable

© 2025 Murali S et al. *JAMA Network Open*.

Source, year	Country	Study design	Age[a]	Gender[a]	Ethnicity[a]	Type of illness	Sample size[b]	Mode of data collection	Prior exposure to ACP/AD/other forms of EOL care planning or discussions	ACP intervention between assessments (if any)
						minute walk distance less than 250 m, or left ventricular assist device or implantable cardioverter defibrillator implant; or Child's class C cirrhosis or Model for End-Stage Liver Disease score greater than 17; or dialysis-dependent renal failure and diabetes; or age 75 years or older and 1 or more life-limiting chronic illnesses; or age 90 years or older; or hospitalization in the past 18 months with a life-limiting illness; or Charlson				

© 2025 Murali S et al. *JAMA Network Open*.

Source, year	Country	Study design	Age[a]	Gender[a]	Ethnicity[a]	Type of illness	Sample size[b]	Mode of data collection	Prior exposure to ACP/AD/other forms of EOL care planning or discussions	ACP intervention between assessments (if any)
						comorbidity score of 6 or higher				
Doorne et al., 2021[5]	Netherlands	Secondary analyses of control group in randomised controlled trial	Mean: 80.7; SD: 8.4	Male - 43.3%; Female - 56.7%	Not reported	Older patients with palliative care needs acutely admitted in hospital in the pulmonary, gastroenterology, oncology , general medicine departments	65	Questionnaire (method of administration not reported)	Not reported	Not applicable
Feifer et al., 2024[6]	USA	Pilot randomised controlled trial	Mean: 32.6; SD: 6.0	Male - 42.2%; Female - 57.8%	White - 84.4%; Asian - 4.4%; Black or african american - 2.2%; More than one race or other - 8.8%	Advanced cancer	45	Telephone questionnaire	55.6% (ACP)	Not applicable
Golombek et al., 2024[7]	Germany	Prospective observational study	Mean: 64.3; SD: 12.9	Male - 43.3%; Female - 56.7%	Not reported	Incurable cancer	34	Questionnaire (method of administration not reported)	76.1% (AD) 61.2% (healthcare proxy)	Not applicable

© 2025 Murali S et al. *JAMA Network Open*.

Source, year	Country	Study design	Age[a]	Gender[a]	Ethnicity[a]	Type of illness	Sample size[b]	Mode of data collection	Prior exposure to ACP/AD/other forms of EOL care planning or discussions	ACP intervention between assessments (if any)
Houben et al., 2017[8]	Netherlands	Secondary analyses of a prospective cohort study	Mean: 67.2; SD: 13.1	Male - 64.1%; Female - 35.9%	Not reported	Global Initiative for Chronic Obstructive Lung Disease, stage III or IV, congestive heart failure with New York Health Associaton classification III and IV symptoms, end-stage chronic renal failure requiring dialysis	206	In-person questionnaire	Not reported	Not applicable
Jabbarian et al., 2019[9]	USA	Prospective cohort study	Mean: 61.3; SD: 8.7	Male - 30.0%; Female - 70.0%	White - 85.6%; Others - 14.4%	Stage IV gastrointestinal, lung, or gynecologic cancer, incurable and poor-prognosis stage III cancers (e.g., pancreas and lung)	104	In-person or telephone questionnaire	Not reported	Not applicable
Knoepke et al., 2022[10]	USA	Secondary analyses of a	Mean: 63.3; SD: 9.8	Male - 85.3%;	White non-hispanic -	End-stage heart failure	158	In-person, telephone, or	Not reported	Formal LVAD education

© 2025 Murali S et al. *JAMA Network Open*.

Source, year	Country	Study design	Age[a]	Gender[a]	Ethnicity[a]	Type of illness	Sample size[b]	Mode of data collection	Prior exposure to ACP/AD/other forms of EOL care planning or discussions	ACP intervention between assessments (if any)
		randomised controlled trial		Female - 14.7%	81.2%; Black - 13.1%; Others - 5.7%			mailed questionnaire		
Kobewka et al., 2021[11]	Canada	Randomised controlled trial	Mean: 78.0 (9.0)	Male – 61.0%	White - 85.0%; East Indian - 3.0%; African / Black North American - 1.0%; Asian / Pacific Islander – 0; First Nation - 1.0%; Others - 10%	Patients admitted to internal medicine and neurology wards	81	In-person questionnaire	14.0% (ACP discussion with family, friends, or physicians) 3.0% (ACP documented)	Not applicable
LoCastro et al., 2023[12]	USA	Pilot single-arm trial	Mean: 76.0; SD: 8.0	Male - 46.7%; Female - 53.3%	White - 93.3%; Black - 6.7%	Older adults with new acute myeloid leukemia diagnosis	15	In-person or telephone questionnaire	Not reported	UR-GOAL communication tool (including an (1) AML educational video, (2) geriatric

© 2025 Murali S et al. *JAMA Network Open*.

Source, year	Country	Study design	Age[a]	Gender[a]	Ethnicity[a]	Type of illness	Sample size[b]	Mode of data collection	Prior exposure to ACP/AD/other forms of EOL care planning or discussions	ACP intervention between assessments (if any)
										assessment, (3) preference elicitation using Best-Worst Scaling, and (4) prognostic awareness)
Malhotra et al., 2020[13]	Singapore	Secondary analyses of a randomised controlled trial	Mean: 64.4; SD: 13.0	Male - 79.0% Female - 21.0%	Chinese - 58.5%; Non-chinese - 41.5%	Heart failure with New York Health Association classification III and IV symptoms	200	In-person questionnaire	24.5% (ACP)	Formal ACP programme
Malhotra et al., 2021[14]	Singapore	Secondary analyses of a randomised controlled trial	Mean: 64.4; SD: 13.0	Male - 79.0%; Female - 21.0%	Chinese - 58.5%; Non-chinese - 41.5%	Heart failure with New York Health Association classification III and IV symptoms	200	In-person questionnaire	25.0% (ACP)	Formal ACP programme
Malhotra et al., 2021[15]	Singapore	Secondary analyses of a prospective cohort study	Median: 61; IQR: 22-92	Male - 45.3%; Female - 54.7%	Chinese - 79.0%; Malay - 13.5%; Indian and other - 7.5%	Stage IV solid malignancy	466	In-person questionnaire	9.0% (ACP/AD)	Not applicable

© 2025 Murali S et al. *JAMA Network Open*.

Source, year	Country	Study design	Age[a]	Gender[a]	Ethnicity[a]	Type of illness	Sample size[b]	Mode of data collection	Prior exposure to ACP/AD/other forms of EOL care planning or discussions	ACP intervention between assessments (if any)
Modes et al., 2022[16]	USA	Secondary analyses of a randomised controlled trial	Median: 76.5; IQR: 17.8	Male - 51.2%; Female - 48.8%	White and non-hispanic - 81.7%; Racial or ethnic minority - 18.3%	Metastatic cancer or inoperable lung cancer; or COPD with forced expiratory volume in 1 second (FEV1) values below 35% of that predicted or oxygen dependence, restrictive lung disease with a total lung capacity below 50% of that predicted, or cystic fibrosis with FEV 1 below 30% of that predicted; or New York Heart Association class III or IV heart failure, pulmonary arterial hypertension with a 6-	252	Telephone or mailed questionnaire	Not reported	Jumpstart-Tips communication-priming intervention

© 2025 Murali S et al. *JAMA Network Open*.

Source, year	Country	Study design	Age[a]	Gender[a]	Ethnicity[a]	Type of illness	Sample size[b]	Mode of data collection	Prior exposure to ACP/AD/other forms of EOL care planning or discussions	ACP intervention between assessments (if any)
						minute walk distance less than 250 m, or left ventricular assist device or implantable cardioverter defibrillator implant; or Child's class C cirrhosis or Model for End-Stage Liver Disease score greater than 17; or dialysis-dependent renal failure and diabetes; or age 75 years or older and 1 or more life-limiting chronic illnesses; or age 90 years or older; or hospitalization in the past 18 months with a life-limiting illness; or Charlson				

© 2025 Murali S et al. *JAMA Network Open*.

Source, year	Country	Study design	Age[a]	Gender[a]	Ethnicity[a]	Type of illness	Sample size[b]	Mode of data collection	Prior exposure to ACP/AD/other forms of EOL care planning or discussions	ACP intervention between assessments (if any)
						comorbidity score of 6 or higher				
Patzer et al., 2018[17]	USA	Randomised controlled trial	Mean: 50.1; SD: 10.3	Male - 61.8%; Female – 38.2%	Black - 45.2%; White non-hispanic - 36.9%; White hispanic - 10.1%; Other - 7.4%	End-stage renal disease (ESRD)	205	Questionnaire (method of administration not reported)	Not reported	Not applicable (focused on control group)
Singh et al., 2023[18]	USA	Pilot single-arm trial	Mean: 73.1; SD: 4.9	Male - 56.5%; Female - 43.5%	American indian or alaska native – 0; Asian - 4.3%; Black or african american – 0; Hispanic or latino - 4.3%; Native hawaiian or other pacific	Solid tumour malignancy	18	In-person or telephone questionnaire	83.3% (MDPOA[c])	Palliative care social work intervention (ALIGN)

© 2025 Murali S et al. *JAMA Network Open*.

Source, year	Country	Study design	Age[a]	Gender[a]	Ethnicity[a]	Type of illness	Sample size[b]	Mode of data collection	Prior exposure to ACP/AD/other forms of EOL care planning or discussions	ACP intervention between assessments (if any)
					islander – 0; White - 87.0%; Other - 4.3%					
Snaman et al., 2024[19]	USA	Pilot randomised controlled trial	Mean: 33.0; SD: 6.1	Male - 40.0%; Female - 60.0%	White - 84.0%; Asian - 6.0%; Black or african american - 2.0%; More than one race - 4.0%; Other - 4.0%	Advanced cancer	38 (only among those who selected life-prolonging care pre-intervention)	Telephone questionnaire	Not reported	10-minute ACP video decision aid
Song et al., 2024[20]	USA	Secondary analyses of a randomised controlled trial	Mean: 59.2; SD: 11.9	Male - 50.9%; Female - 49.1%	Non-black – 17.3%; Black – 82.7%	Kidney failure patients on chronic dialysis	110	Telephone questionnaire	13.6% (AD)	Usual care - Usual ACP discussion<br><br>Intervention - ACP psychoeducational intervention Sharing the Patient's Illness

© 2025 Murali S et al. *JAMA Network Open*.

Source, year	Country	Study design	Age[a]	Gender[a]	Ethnicity[a]	Type of illness	Sample size[b]	Mode of data collection	Prior exposure to ACP/AD/other forms of EOL care planning or discussions	ACP intervention between assessments (if any)
										Representations to Increase Trust (SPIRIT)
Srinivas et al., 2024[21]	USA	Retrospective cohort study (chart review)	Median: 61; IQR: 52-57	Male - 50.4%; Female - 49.6%	White - 82.4%; Non-white - 17.6%	Requiring an emergency general surgery (exploratory laparotomy)	484	Electronic medical records	Not reported	Not applicable
Stegmann et al., 2021[22]	Netherlands	Descriptive qualitative study of intervention group from a randomised controlled trial	Mean: 75.3; SD: 6.4	Male - 82.8%; Female - 17.2%	Not reported	Non-curable cancer	29	In-person questionnaire and medical records	Not reported	Not applicable
Valentino et al., 2023[23]	Brazil	Prospective cohort study	Mean: 59.3; SD: 13.1		Not reported	Advanced cancer with a performance status ≤3 according to Eastern Cooperative Oncology Group (ECOG) and a life expectancy between 3 and 12 months	112	In-person questionnaire	Not reported	Not applicable

© 2025 Murali S et al. *JAMA Network Open*.

Source, year	Country	Study design	Age[a]	Gender[a]	Ethnicity[a]	Type of illness	Sample size[b]	Mode of data collection	Prior exposure to ACP/AD/other forms of EOL care planning or discussions	ACP intervention between assessments (if any)
van der Velden et al., 2021[24]	Netherlands	Secondary analyses of a randomised controlled trial	Mean: 63.5; SD: 10.1	Male - 51.4%; Female - 48.6%	Not reported	Metastatic/inoperable tumour, indicated by a median life expectancy of < 12 months without anticancer treatment and a median survival benefit of < 6 months	129	Mailed questionnaire	Not reported	Communication aid for patients to stimulate shared decision making about palliative systemic treatment (CHOICE); but arms were not distinguished in this paper
Yun et al., 2019[25]	South Korea	Randomised controlled trial	Mean: 57.1; SD: 11.0	Male - 38.0%; Female – 62.0%	Not reported	Advanced cancer	81	In-person questionnaire	14.0% (ACP discussion with family, friends, or physicians) 3.0% (ACP documented)	Not applicable (focused on control group)

[a]Distribution at baseline
[b]Sample size of patients assessed for unstable goals/preferences over time for whom data to obtain proportion of patients that changed goals/preferences at least once over time was available
[c]Note: MDPOA = Medical Durable Power of Attorney

© 2025 Murali S et al. *JAMA Network Open*.

eTable 3. Rating of study quality

Study ID	Author	Year	1	2	3	4	5	6	7	8	9	10	11	12	13	14	Quality score	Quality rating
1	Auriemma et al.[1]	2024	Y	Y	NA	Y	N	Y	Y	Y	Y	N	Y	NA	NA	N	8/11 (72.7%)	Fair
2	Benton et al.[2]	2015	Y	Y	NA	Y	N	NA	NA	NA	NA	NA	Y	NA	NA	NA	4/5 (80.0%)	Good
3	Campos et al.[3]	2022	Y	Y	NA	Y	N	NA	NA	NA	NA	NA	Y	NA	NA	NA	4/5 (80.0%)	Good
4	Curtis et al.[4]	2018	Y	Y	Y	Y	Y	NA	NA	NA	NA	NA	Y	NA	N	NA	6/7 (85.7%)	Good
5	Doorne et al.[5]	2021	Y	Y	NR	Y	N	Y	Y	Y	Y	N	Y	NA	N	Y	9/13 (69.2%)	Fair
6	Feifer et al.[6]	2024	Y	Y	N	Y	Y	NA	NA	NA	NA	NA	Y	NA	Y	NA	6/7 (85.7%)	Fair
7	Golombek et al.[7]	2024	Y	Y	N	Y	N	Y	Y	Y	0.5Y	0.5Y	Y	NA	N	N	8/13 (61.5%)	Fair
8	Houben et al.[8]	2017	Y	Y	NR	Y	N	Y	Y	Y	Y	N	Y	NA	N	N	8/13 (61.5%)	Fair
9	Jabbarian et al.[9]	2019	Y	Y	Y	Y	N	Y	Y	Y	Y	N	Y	NA	N	N	9/13 (69.2%)	Fair
10	Knoepke et al.[10]	2022	Y	Y	Y	Y	Y	Y	Y	Y	Y	Y	Y	NA	N	Y	12/13 (92.3%)	Good
11	LoCastro et al.[12]	2023	Y	Y	NR	Y	N	Y	Y	Y	0.5Y	NA	Y	NA	N	N	7.5/12 (62.5%)	Fair
12	Malhotra et al.[13]	2020	Y	Y	NR	Y	Y	Y	Y	Y	Y	Y	Y	NA	N	Y	11/13 (84.6%)	Good
13	Malhotra et al.[14]	2021	Y	Y	NR	Y	Y	Y	Y	Y	Y	Y	Y	NA	N	Y	11/13 (84.6%)	Good
14	Malhotra et al.[15]	2021	Y	Y	Y	Y	Y	Y	Y	Y	Y	Y	Y	NA	N	Y	12/13 (92.3%)	Good
15	Modes et al.[16]	2022	Y	Y	Y	Y	Y	NA	NA	NA	NA	NA	Y	NA	N	NA	6/7 (85.7%)	Good
16	Patzer et al.[17]	2018	Y	Y	Y	Y	Y	Y	Y	NA	Y	NA	Y	NA	Y	N	10/11 (91.0%)	Good
17	Singh et al.[18]	2023	Y	Y	Y	Y	N	NA	NA	NA	NA	NA	Y	NA	N	NA	5/7 (71.4%)	Fair
18	Snaman et al.[19]	2024	Y	Y	N	Y	Y	NA	NA	NA	NA	NA	Y	NA	Y	NA	6/7 (85.7%)	Good
19	Song et al.[20]	2024	Y	Y	Y	Y	Y	Y	Y	Y	Y	NA	Y	NA	N	Y	11/12 (91.7%)	Good
20	Srinivas et al.[21]	2024	Y	Y	NA	Y	N	Y	Y	Y	Y	N	Y	NA	NA	Y	9/11 (81.9%)	Good
21	Stegmann et al.[22]	2021	Y	Y	NA	Y	N	NA	NA	NA	NA	NA	Y	NA	NA	NA	4/5 (80.0%)	Good
22	Valentino et al.[23]	2023	Y	Y	Y	Y	N	NA	NA	NA	NA	NA	Y	NA	N	NA	5/7 (71.4%)	Fair
23	van der Velden et al.[24]	2021	Y	Y	Y	Y	Y	Y	Y	Y	Y	Y	Y	NA	N	Y	12/13 (92.3%)	Fair
24	Yun et al.[25]	2019	Y	Y	Y	Y	Y	Y	Y	NA	Y	NA	Y	NA	N	N	9/11 (81.9%)	Good
25	Kobewka et al. [11]	2021	Y	Y	N	Y	Y	NA	NA	NA	NA	NA	Y	NA	Y	NA	6/7 (85.7%)	Good

© 2025 Murali S et al. *JAMA Network Open.*

eReferences.

1. Auriemma CL, Song A, Walsh L, et al. Classification of Documented Goals of Care Among Hospitalized Patients with High Mortality Risk: a Mixed-Methods Feasibility Study. *Journal of General Internal Medicine.* 2024;39(10):1839-1849.
2. Benton K, Stephens J, Vogel R, et al. The Influence of Race on End-of-Life Choices Following a Counselor-Based Palliative Consultation. *American Journal of Hospice and Palliative Medicine.* 2015;32(1):84-89.
3. Campos E, Isenberg SR, Lovblom LE, et al. Supporting the Heterogeneous and Evolving Treatment Preferences of Patients With Heart Failure Through Collaborative Home-Based Palliative Care. *Journal of the American Heart Association.* 2022;11(19).
4. Curtis JR, Downey L, Back AL, et al. Effect of a patient and clinician communication-priming intervention on patient-reported goals-of-care discussions between patients with serious illness and clinicians: A randomized clinical trial. *JAMA Internal Medicine.* 2018;178(7):930-940.
5. Van Doorne I, Van Rijn M, Dofferhoff SM, Willems DL, Buurman BM. Patients' preferred place of death: Patients are willing to consider their preferences, but someone has to ask them. *Age and Ageing.* 2021;50(6):2004-2011.
6. Feifer D, Helton G, Wolfe J, Volandes A, Snaman JM. Adolescents and young adults with cancer conversations following participation in an advance care planning video pilot. *Supportive Care in Cancer.* 2024;32(3).
7. Golombek T, Hegewald N, Schnabel A, Fries H, Lordick F. Stability of End-of-Life Care Wishes and Gender-Specific Characteristics of Outpatients with Advanced Cancer under Palliative Therapy: A Prospective Observational Study. *Oncology Research and Treatment.* 2024;47(5):189-197.
8. Houben CHM, Spruit MA, Schols JMGA, Wouters EFM, Janssen DJA. Instability of Willingness to Accept Life-Sustaining Treatments in Patients With Advanced Chronic Organ Failure During 1 Year. *Chest.* 2017;151(5):1081-1087.
9. Jabbarian LJ, Maciejewski RC, Maciejewski PK, et al. The Stability of Treatment Preferences Among Patients With Advanced Cancer. *Journal of Pain and Symptom Management.* 2019;57(6):1071-1079.e1071.
10. Knoepke CE, Chaussee EL, Matlock DD, et al. Changes over Time in Patient Stated Values and Treatment Preferences Regarding Aggressive Therapies: Insights from the DECIDE-LVAD Trial. *Medical Decision Making.* 2022;42(3):404-414.
11. Kobewka D, Heyland DK, Dodek P, et al. Randomized Controlled Trial of a Decision Support Intervention About Cardiopulmonary Resuscitation for Hospitalized Patients Who Have a High Risk of Death. *J Gen Intern Med.* 2021;36(9):2593-2600.
12. LoCastro M, Wang Y, Sanapala C, et al. Patient preferences, regret, and health-related quality of life among older adults with acute myeloid leukemia: A pilot longitudinal study. *Journal of Geriatric Oncology.* 2023;14(5).
13. Malhotra C, Hu M, Malhotra R, et al. Instability in End-of-Life Care Preference Among Heart Failure Patients: Secondary Analysis of a Randomized Controlled Trial in Singapore. *J Gen Intern Med.* 2020;35(7):2010-2016.
14. Malhotra C, Bundoc FG, Sim D, Jaufeerally FR, Finkelstein EA. Instability in Preference for Place of Death Among Patients With Symptoms of Advanced Heart Failure. *J Am Med Dir Assoc.* 2021;22(2):349.e329-349.e334.
15. Malhotra C, Koh LE, Teo I, Ozdemir S, Chaudhry I, Finkelstein E. A Prospective Cohort Study of Stability in Preferred Place of Death Among Patients With Stage IV Cancer in Singapore. *J Natl Compr Canc Netw.* 2021;20(1):20-28.
16. Modes ME, Engelberg RA, Nielsen EL, et al. Seriously Ill Patients’ Prioritized Goals and Their Clinicians’ Perceptions of Those Goals. *Journal of Pain and Symptom Management.* 2022;64(4):410-418.
17. Patzer RE, McPherson L, Basu M, et al. Effect of the iChoose Kidney decision aid in improving knowledge about treatment options among transplant candidates: A randomized controlled trial. *Am J Transplant.* 2018;18(8):1954-1965.
18. Singh S, Dafoe A, Lahoff D, et al. Pilot Trial of a Social Work Intervention to Provide Palliative Care for Adults with Cancer in Skilled Nursing Facilities. *J Palliat Med.* 2023;26(4):527-538.
19. Snaman JM, Feifer D, Helton G, et al. A Pilot Randomized Trial of an Advance Care Planning Video Decision Support Tool for Adolescents and Young Adults With Advanced Cancer. *J Natl Compr Canc Netw.* 2023;21(7):715-723.e717.
20. Song M-K, Paul S, Pelkmans J, Ward SE. Pandemic Effects on Stability of End-of-Life Preferences and Patient-Surrogate Dyad Congruence. *Journal of Pain and Symptom Management.* 2024;67(6):571-579.e572.
21. Srinivas S, Villarreal ME, Baselice H, et al. Identifying Factors Associated With Code Status Changes After Emergency General Surgery. *Journal of Surgical Research.* 2024;294:150-159.

© 2025 Murali S et al. *JAMA Network Open*.

22. Stegmann ME, Brandenbarg D, Reyners AKL, van Geffen WH, Hiltermann TJN, Berendsen AJ. Treatment goals and changes over time in older patients with non-curable cancer. *Support Care Cancer.* 2021;29(7):3849-3856.
23. Valentino TCdO, Paiva CE, de Oliveira MA, et al. Preference and actual place-of-death in advanced cancer: prospective longitudinal study. *BMJ Supportive & Palliative Care.* 2024;14(e1):e1402-e1412.
24. van Doorne I, van Rijn M, Dofferhoff SM, Willems DL, Buurman BM. Patients' preferred place of death: patients are willing to consider their preferences, but someone has to ask them. *Age Ageing.* 2021;50(6):2004-2011.
25. Yun YH, Kang E, Park S, et al. Efficacy of a Decision Aid Consisting of a Video and Booklet on Advance Care Planning for Advanced Cancer Patients: Randomized Controlled Trial. *Journal of Pain and Symptom Management.* 2019;58(6):940-948.e942.

© 2025 Murali S et al. *JAMA Network Open*.
