# Supplementary material for: Instability in End-of-Life Goals and Preferences of Patients Who Are Seriously Ill: A Systematic Review
Source: JAMA Netw Open. 2025 Nov 14;8(11):e2541264. doi: 10.1001/jamanetworkopen.2025.41264 (PMC12619100; doi:10.1001/jamanetworkopen.2025.41264)
Supplement: Supplement 2. — Data Sharing Statement [file jamanetwopen-e2541264-s002.pdf]

Data Sharing Statement

Murali. Instability in End-of-Life Goals and Preferences of Patients Who Are Seriously Ill. *JAMA Netw Open*. Published November 14, 2025. doi:10.1001/jamanetworkopen.2025.41264

Data
Data available: No

Additional Information
Explanation for why data not available: This systematic review does not include original data. Data are extracted from the literature and are publicly available.
